# Unexpected Role of α-Fetoprotein in Spermatogenesis

**DOI:** 10.1371/journal.pone.0019387

**Published:** 2011-05-04

**Authors:** Futoshi Yazama, Akihiro Tai

**Affiliations:** Department of Life Sciences, Prefectural University of Hiroshima, Shobara City, Hiroshima, Japan; University of South Florida College of Medicine, United States of America

## Abstract

**Background:**

Heat shock severely affects sperm production (spermatogenesis) and results in a rapid loss of haploid germ cells, or in other words, sperm formation (spermiogenesis) is inhibited. However, the mechanisms behind the effects of heat shock on spermatogenesis are obscure.

**Methodology/Principal Findings:**

To identify the inhibitory factor of spermiogenesis, experimental cryptorchid (EC) mice were used in this study. Here we show that α-fetoprotein (AFP) is specifically expressed in the testes of EC mice by proteome analysis. AFP was also specifically localized spermatocytes by immunohistochemical analysis and was secreted into the circulation system of EC mice by immunoblot analysis. Since spermatogenesis of an advanced mammal cannot be reproduced with *in vitro*, we performed the microinjection of AFP into the seminiferous tubules of normal mice to determine whether AFP inhibits spermiogenesis *in vivo*. AFP was directly responsible for the block in spermiogenesis of normal mice. To investigate whether AFP inhibits cell differentiation in other models, using EC mice we performed a partial hepatectomy (PH) that triggers a rapid regenerative response in the remnant liver tissue. We also found that liver regeneration is inhibited in EC mice with PH. The result suggests that AFP released into the blood of EC mice regulates liver regeneration by inhibiting the cell division of hepatocytes.

**Conclusions/Significance:**

AFP is a well-known cancer-specific marker, but AFP has no known function in healthy human beings. Our findings indicate that AFP expressed under EC conditions plays a role as a regulatory factor in spermatogenesis and in hepatic generation.

## Introduction

In most mammals, testes are kept about 5°C below body temperature, and this is the reason for their descent into the scrotum. Cooling of testes depends, in part, on perspiration and evaporative heat loss from the surface of the scrotum. Heat shock, a slight increase in temperature for a short period of time, and cryptorchidism, which is more convenient for longer periods, severely affect sperm production (spermatogenesis) and result in a rapid loss of haploid germ cells, or in other words, sperm formation (spermiogenesis) is inhibited [1], [2]. The existence of a functional barrier between the blood and lymph systems and the interior of the seminiferous tubule is well established [3], [4]. The blood-testis barrier (BTB) does not change morphologically and the integrity of the BTB is not affected in experimental cryptorchidism (EC) [5], [6]. There are no haploid germ cells in the seminiferous epithelium in mice 14 days after the cryptorchid operation (EC 14 Ds) [6]. These facts suggest that the loss of haploid germ cells in heat shock may inhibit the differentiation into haploid germ cells from diploid germ cells by an endogenous factor of the testis. In male germ cells, high temperatures induce an increase in the synthesis of several proteins and a decrease in many others [7], [8], [9], [10]. However, the endogenous factor has not been discovered.

## Results

### Proteome analysis of EC testes

**Figure 1:**
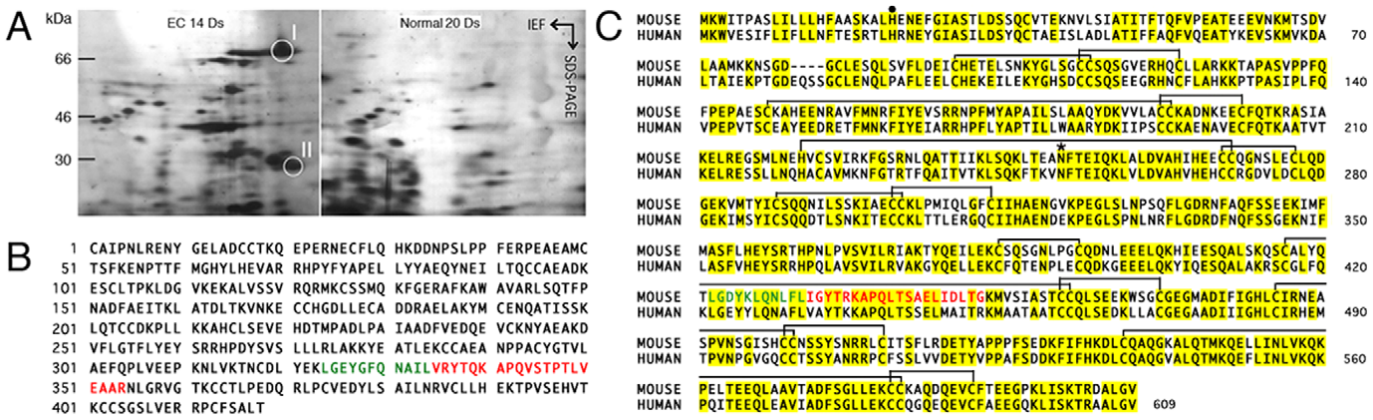
Proteome analysis of EC testes. Silver-stained 2-D gels of testis proteins from EC 14 Ds and normal 20 Ds mice (A). Full-length amino acid sequence of mice AFP (B). Homology of mouse and human AFP (C). Spots I (about 72 kDa) and II (about 26 kDa) are detected in testis proteins from EC 14 Ds (A, white circles). Internal N-terminal amino acid sequence of spot I is highlighted in green and red. Internal N-terminal amino acid sequence of spot II is highlighted in red. The homology of mouse and human AFP is 65% and the matching amino acid residues are shown in yellow. Metal binding site (22; black circle), glycosylation site (251; asterisk) and disulfide bonds (99–114, 113–124, 148–193, 192–201, 224–270, 269–277, 289–303, 384–393, 416–462, 461–472, 485–501, 500–511, 538–583, and 582–591) were completely conserved (C).

To identify the endogenous factor in EC mice testes, subtraction of two-dimensional (2-D) maps of testis proteins extracted from EC 14 Ds mice and normal 20-day post-partum (Ds) mice was performed. Diploid germ cells (spermatogonia and spermatocytes) were present, but there were no haploid germ cells in the seminiferous epithelium in normal 18–20 Ds mice [11]. Significantly, protein spots I (about 72 kDa) and II (about 26 kDa) were present in EC 14 Ds testes, but were missing in normal 20 Ds testes (Fig. 1A). These spots from EC 14 Ds testes were excised, and the internal N-terminal amino acid sequences were determined by mass spectrometry (spot I, LGEYGFQNAILVRYTQKAPQVSTPTLVEAAR, Fig. 1B, highlighted in green and red; spot II, VRYTQKAPQVSTPTLVEAAR, Fig. 1B, highlighted in red) and compared with known sequences using the NCBInr database. Our analysis indicated that spots I and II were identical to mice AFP (Accession Number, AAA37190) and suggested that spot II was likely to be an electrophoretic degradation product of spot I. The alignment of the putative amino acid sequence of mouse AFP (Accession Number, P02772, in UniProtKB) showed strong similarity with human AFP (Accession Number, P02771, in UniProtKB), and the metal binding site, glycosylation site, and disulfide bonds were completely conserved (Fig. 1C).

### Testis specific expression of AFP in EC mice

**Figure 2:**
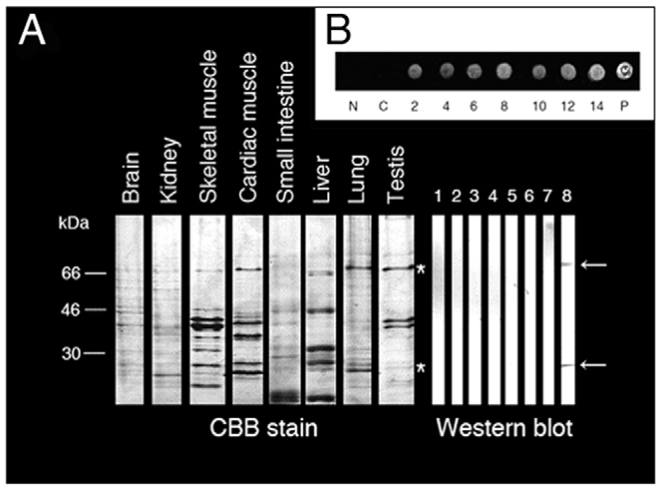
Testis specific expression of AFP in EC mice. SDS-PAGE and Western blotting of multiple tissues reacts using an anti-mice AFP antibody (A). Dot blotting of EC mice sera after different days of cryptorchidism (B). The AFP antibody reacts with two bands (about 72 and 26 kDa) in the testis (A, lane 8, arrows), and no signals are seen in the other tissues. Asterisks indicate the two bands which correspond to the positive bands of Western blot. All of the EC mice sera react positively, but the 35 Ds control sera are negative (C in B). N, negative control, 0.1 mg/ml BSA; P, positive control, 0.1 mg/ml synthetic peptide.

We then examined the testis specific expression of AFP in EC mice. Eight kinds of EC 14 Ds mice tissues (1. brain, 2. kidney, 3. skeletal muscle, 4. cardiac muscle, 5. small intestine, 6. liver, 7. lung, and 8. testis) were used for biochemical analyses. Figure 2 shows multiple tissues of EC 14 Ds mice run on 12% sodium dodecyl sulfate polyacrylamide gel electrophoresis (SDS-PAGE). Proteins were stained with Coomassie Brilliant Blue (CBB) R-250 (A, left) or transferred to nitrocellulose membranes incubated with an anti-mice AFP antibody (A, right, lanes 1–8). Western blotting with an anti-mice AFP antibody showed two bands (about 72 and 26 kDa) in the testis (A, lane 8, arrows), but not in the other tissues. These AFP immunoreactive proteins correspond to the two bands that were visible by CBB in the testis (A, asterisks). For dot blot analysis, blood was collected from EC mice every second day until 14 days after the cryptorchid operation. Dot blotting of EC mice sera with an anti-mice AFP antibody showed a positive reaction in all EC samples (B), but not in the control 35 Ds sera (C in B). These results demonstrate that AFP is produced in testes and released into the blood of EC mice.

### Localization of AFP in EC testicular cells and microinjection of AFP into the seminiferous tubules of normal mice

**Figure 3:**
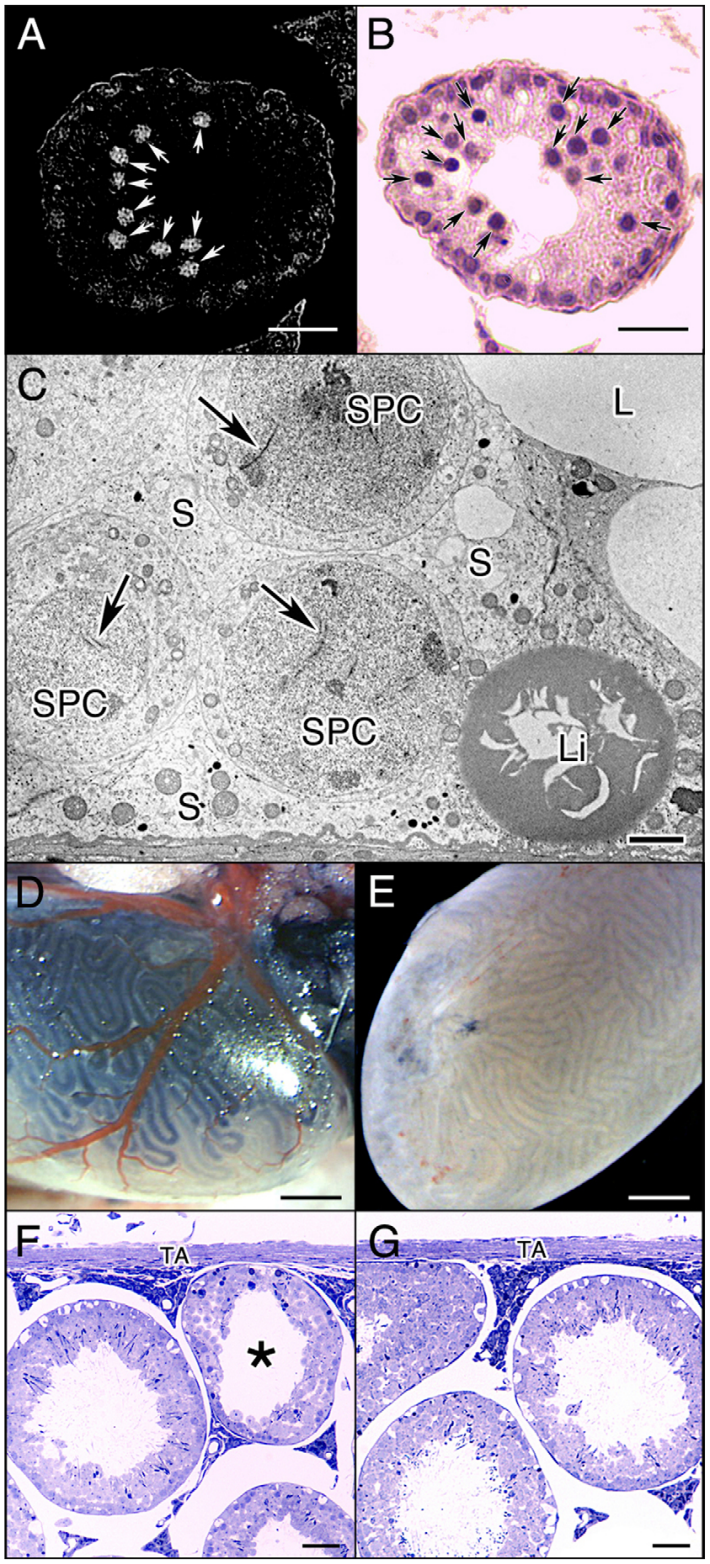
Localization of AFP in EC testicular cells and microinjection of human AFP into the seminiferous tubules of normal mice. Immunohistochemical staining of EC 14 Ds testis (A). Conventional HE stained paraffin sections of EC 14 Ds testis (B). Basal aspect of the seminiferous epithelium of EC 14 Ds mice (C). Microinjection of AFP into the seminiferous tubules through the efferent ducts of normal 49 Ds male mice (D). Fixed testes five days after the microinjection (E). AFP microinjected testis (F). BSA microinjected testis (G). White arrows indicate AFP positive cells (A), and black arrows indicate spermatocytes (B). Scale bar, 50 µm. In the pachytene stage of the EC 14 Ds mice spermatocytes, synaptonemal complexes were clearly seen (C, arrows). Scale bar, 2 µm. SPC, spermatocyte; S, Sertoli cells; L, lumen of seminiferous tubule, Li, lipid droplet. Blue staining of the seminiferous tubules right under the tunica albuginea reflects the success of the microinjection (D). Scale bar, 1 mm. Five days after the microinjection, fixed testes containing faint blue staining of the seminiferous tubles right under the tunica albuginea (E). Scale bar, 1 mm. Semithin sections were stained with 1% toluidine blue dye (F and G). No evidence of spermiogenesis is observed in the shrunken seminiferous tubule of AFP microinjected testis (asterisk in F), but normal appearing spermatogenesis is observed in the BSA microinjected testis (G). Scale bar, 50 µm. TA, tunica albuginea.

Next we decided to investigate AFP for expression in the testicular cells of EC 14 Ds testes using immunohistochemical techniques. AFP-positive cells were observed in the EC 14 Ds seminiferous tubules (Fig. 3A, white arrows), and were comparatively large and situated apart from the testicular lamina propria, which corresponded to spermatocytes in conventional paraffin sections stained with hematoxylin and eosin (Fig. 3B, black arrows). Figure 3C shows basal aspect of the seminiferous epithelium of EC 14 Ds mice, the pachytene stage of spermaticytes was clearly seen.

To determine whether AFP inhibits cell division during spermatogenesis *in vivo*, we performed the microinjection of purified human AFP derived from umbilical sera as a model protein into the lumen of the seminiferous tubules through the efferent ducts of normal 49 Ds male mice. Blue staining of the seminiferous tubules right under the tunica albuginea reflect the success of the microinjection (Fig. 3D). Although the blue stain discolors, seminiferous tubules right under the tunica albuginea dyed to a faint blue can be confirmed with the naked eye in the fixed testes five days after the microinjection (Fig. 3E). Therefore, small pieces of fixed testis tissue containing faint blue stained seminiferous tubules with the tunica albuginea were post-fixed and embedded in a resin. Semithin sections were stained with 1% toluidine blue dye and examined under a light microscope (Fig. 3F and G). No evidence of spermiogenesis is observed in the shrunken seminiferous tubule in the AFP microinjected testis (Fig. 3F, asterisk). Normal appearing spermatogenesis is observed in the bovine serum albumin (BSA) microinjected testis (Fig. 3G). The morphology of the shrunken seminiferous tubule in EC mice (Fig. 3B) and AFP microinjected mice (Fig. 3F, asterisk) looks the same.

### Partial hepatectomy on control and EC mice

**Figure 4:**
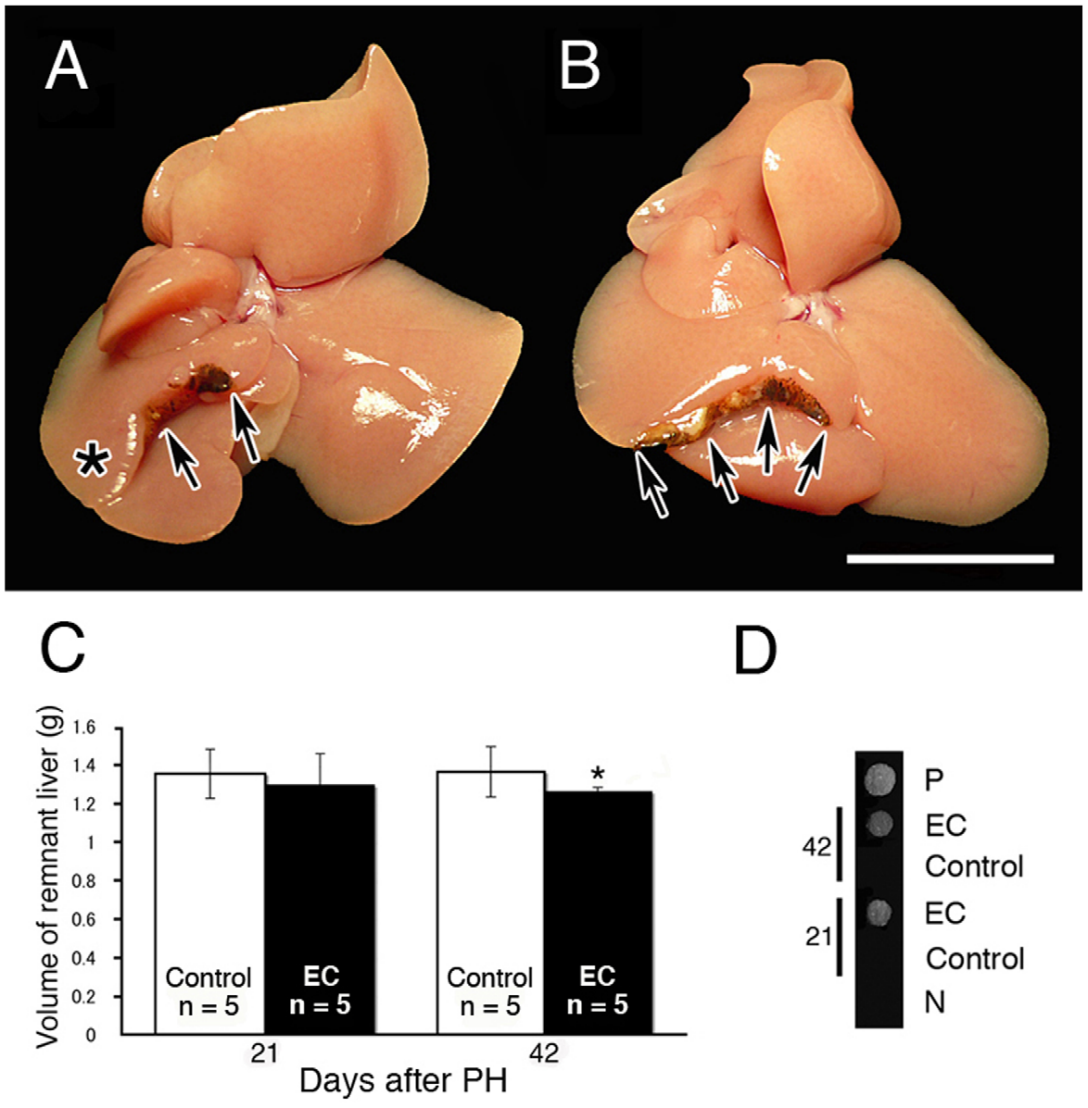
Partial hepatectomy on control and EC mice. Remnant liver of control mice 35 days after PH (A). Remnant liver of EC mice 35 days after PH (B). Scale bar, 1 cm. Volume of remnant liver in control and EC mice 21 and 42 days after PH (C). Dot blotting of control and EC mice sera 21 and 42 days after PH reacted using an anti-mice AFP antibody (D). Quantification of the remnant liver volume in EC mice 42 days after PH shows a statistically significant low value (C, *p = 0.05 by unpaired *t* test). Data are presented as mean ± SD. EC mice sera reacted positively, but the control sera are negative (D). N, negative control, 0.1 mg/ml BSA; P, positive control, pregnant mice sera.

Additionally, to determine whether AFP inhibits cell differentiation during liver regeneration, we performed a partial hepatectomy (PH) on EC mice as another model of regenerative differentiation. Figure 4A and B show the remnant liver of control and EC mice 35 days after PH. The asterisk indicates the regenerated liver tissue (Fig. 4A), and the arrows indicate the edge of PH (Fig. 4A, B). Quantification of the remnant liver volume in EC mice 42 days after PH shows a statistically significant low value (Fig. 4C, asterisk). Dot blotting of control and EC mice sera with an anti-mice AFP antibody (sc-8108) shows a positive reaction in the EC samples, but not in the control sera (Fig. 4D). These results demonstrate that liver regeneration is inhibited in EC mice with PH.

## Discussion

Most adult organs do not have the capacity for regeneration, thus the unique capability of the liver to regenerate after resection is most fascinating. A model of hepatic resection is essential to the study of liver regeneration and its effects on various pathological processes [12], [13], [14]. The fact that testes also regenerate is off the beaten path, though liver regeneration is well known. Heat shock severely affects spermatogenesis and results in a rapid loss of haploid germ cells [1], [2]. No one knows why testes cannot function at body temperature in mammals that normally have scrotal testes. This appears to be due to the higher temperature in the abdomen, because if a heated testis is cooled but left in the abdomen, spermatogenesis will proceed normally. This process is called regenerative differentiation of the testis. As such, the only organs with regenerative differentiation in adults are the liver and the testis.

In this paper we show that the protein most significantly up-regulated in EC 14 Ds testes was the well-known cancer-specific marker AFP (Fig. 1). AFP produced in organs other than testes was not delivered to spermatocytes because the integrity of the BTB was not affected in EC 14 Ds mice [6]. Therefore, AFP might be produced in spermatocytes under conditions of heat shock. This fact suggests that the loss of haploid germ cells may inhibit differentiation into haploid germ cells from diploid germ cells, potentially due to the up-regulation of AFP expression in the pachytene stage of spermatocytes under EC conditions.

In present biotechnology, meiosis of an advanced mammal cannot be reproduced with *in vitro*, though spermatocytes can be cultured [15], [16], [17]. Additionally, because the integrity of the BTB was not affected in EC mice, AFP injected through the blood vessels was not delivered to spermatocytes [6]. Therefore, to determine whether AFP inhibits cell division during spermatogenesis *in vivo*, we performed the microinjection of purified human AFP derived from umbilical sera into the lumen of the seminiferous tubules through the efferent ducts of normal mice. Because the adequate dose of AFP was not understood, a high dose of purified human AFP was microinjected into the seminiferous tubules of normal male mice. As shown in Fig. 3D, also in the same seminiferous tubules the blue staining was a different tone according to the domain of the seminiferous tubules. Therefore, we thought that even if a constant dose of AFP was microinjected into the lumen of the seminiferous tubules, the concentration gradient of AFP was caused by the domain of the seminiferous tubules. The blue stain of the seminiferous tubules right under the tunica albuginea faded completely seven days after the microinjection (data not shown). We fixed the testes five days after the microinjection because we were unable to confirm the domain where the microinjection had succeeded seven days later (Fig. 3E). Because the alignment of the putative amino acid sequence of mouse AFP showed strong similarity with human AFP, the metal binding site, glycosylation site, and disulfide bonds were completely conserved (Fig. 1C). We believe that the function and structure of mouse and human AFP are probably the same. We also believe that spermiogenesis must be inhibited if an adequate dose of AFP is delivered from the lumen of the seminiferous tubules to spermatocytes. Thus human AFP was directly responsible for the block in spermiogenesis of normal mice five days after the microinjection (Fig. 3F). In contrast to AFP, normal appearing spermatogenesis is observed in the testis microinjected with a high dose of BSA (Fig. 3G). In the case of EC, spermatogenesis will proceed normally again by testes migrating back to the scrotum, and we do not know whether the inhibition of spermiogenesis induced by AFP microinjection is reversible or not because the trypan blue stain fades completely seven days after the microinjection. Therefore we were unable to confirm the domain where the microinjection had succeeded in this experiment. To determine whether AFP inhibits cell division during liver regeneration, we performed PH that triggers a rapid regenerative response in the remnant liver tissue in EC mice as another model of regenerative differentiation. We also found that liver regeneration is inhibited in EC mice with PH (Fig. 4). This result suggests that AFP released into the blood of EC mice regulates liver regeneration by inhibiting the cell division of hepatocytes in the remnant liver tissue in EC mice. In fact, physical liver regeneration was rare in PH excising about 20% volume of the liver of control mice (Fig. 4A), and the majority of liver regeneration might be due to vicarious hypertrophy of remnant hepatocytes in the control mice.

Analysis of the expression of AFP during liver development in rodent and human embryos is of special interest because this protein is present only in very small amounts in the adult liver, but expressed in relatively large quantities during hepatocarcinogenesis and in most neoplasms [18]. Generally speaking, if an individual has an elevated level of AFP, a search for testicular or liver cancer will be made, but AFP has no known function in healthy human beings. The testis is an important organ because it produces spermatozoa. Under a high temperature condition, the testis might not be able to produce normal mature sperm. If abnormal genetic information is passed onto the next generation, the preservation of species cannot be maintained. Thus AFP might play an important role in the preservation of species by regulating the meiosis of spermatocytes. Although AFP is produced by embryonic hepatocytes [19], [20], [21], the functions of embryonic AFP are also presently unknown. From our findings we speculate that AFP may regulate morphogenesis of the liver by inhibiting increased cell division of hepatocytes in embryos. Therefore, the present study proposes that AFP expressed under EC conditions plays a role as a regulatory factor in spermatogenesis and in hepatic generation. In a future study we will provide molecular mechanisms to show that AFP controls cell division.

## Materials and Methods

### Animals

C57BL/6J male mice (CLEA Japan, Inc.) were used for the EC and PH experiments. The 35 Ds male mice were made bilaterally cryptorchid by surgery. Seven days after the cryptorchid operation we performed PH excising about 20% volume of the liver (about 0.2 g) of EC mice using a radio knife (18000-00; Muromachi Kikai, Tokyo, Japan). As control animals we only performed PH on 42 Ds male mice. Microinjection of human AFP into the lumen of the seminiferous tubules through the efferent ducts of normal 49 Ds ICR male mice (CLEA Japan, Inc.) was carried out using a previously described microinjection protocol [22]. Though the efferent duct is extremely small, it is possible to microinject by hand if skilled using a stereomicroscope, even if there is no special apparatus. Human AFP (#105-10, Lee Biosolutions, Inc., USA) was purified by a PD-10 column (GE Healthcare, UK Ltd.). Purified human AFP (100 µg/ml in PBS containing 0.05% trypan blue stain solution) was injected intratesticularly. As a negative control protein, BSA (100 µg/ml in PBS containing 0.05% trypan blue stain solution) was also injected intratesticularly. Five days after the microinjection, testes were fixed with periodate-lysin-paraformaldehyde (PLP). Small pieces of fixed testis tissue were post-fixed with 1% buffered osmium (pH 7.2) at 4°C for 90 min, dehydrated using an ethanol series and embedded in EPON 812 (TAAB Laboratories, Berkshire, England).

### Proteome analysis of EC testes

Testes were removed surgically from mice that were fully anesthetized with pentbarbital and decapsuled and the major testicular vessel excised. Testes (0.1 g) were homogenized with 1,000 µl lysis buffer (8 M urea in redistilled water containing 2% nonidet P-40, 2% ampholine, 5% ß-mercaptoethanol and 5% polyvinylpyrrolidone). The insoluble materials were removed by centrifugation (20,000 *g* for 30 min at 4°C) and the supernatant (300 µl) was subjected to isoelectric focusing (IEF). In principle, 2D-PAGE was performed as described by O'Farrell [23], but modified to improve the resolution [24]. The first dimension of IEF was run by applying the voltage in a stepwise manner: 200 V for the first 0.5 h, 300 V for the next 0.5 h, 400 V for the next 18 h and, finally 800 V for 1 h. The second dimension of SDS-PAGE was performed as described by Hirano and W-Liebold [25]. After electrophoresis, proteins were detected CBB or silver-stained. In-gel digestion and mass spectrometric identification of proteins were performed by ProPhoenix Co., Ltd. (Higashihiroshima, Japan).

### Immunohistochemical and immunoblot analysis

The synthetic peptide (LGEYGFQNAILVRYTQKAPQVSTPTLVEAAR) and the anti-mice AFP polyclonal antibody were prepared by Operon Biotechnology (Tokyo, Japan) or by Santa Cruz Biotechnology, Inc., USA (sc-8108). Mice were fully anesthetized and perfused with a PLP fixative via the left ventricle at room temperature (RT) and then immersed in the fixative at RT for 4 h [26]. The fixed testes were dehydrated in an ethanol series and embedded in paraffin. Paraffin sections were cut at 5 µm. Immunohistochemical and immunoblot analysis was performed as described previously [27], [28].

The experiments were approved by the Committee for Ethics on Animal Experiments of the Prefectural University of Hiroshima, Japan (ID: 2010-009).
